# Supplementary material for: Transcriptome Analysis of the Hippocampus in Domestic Laying Hens with Different Fear Responses to the Tonic Immobility Test
Source: Animals (Basel). 2025 Jun 26;15(13):1889. doi: 10.3390/ani15131889 (PMC12248666; doi:10.3390/ani15131889)
Supplement: Supplementary file 1 [file animals-15-01889-s001.zip › animals-3671027-supplementary.pdf]

Table S1 Feed Composition Table

Ingredients	Content (%)	Nutritional content indicators	Estimated
Corn	65.2	Metabolizable energy (ME)	~2755 kcal/kg
Soybean meal	20.8	Crude protein (CP)	18.6 - 19.4%
Vegetable oil	1.1	Crude fat	5.6 - 6.4%
Powdered rock	3.4	Calcium (Ca)	3.55 - 3.75%
Calcium hydrogen phosphate	0.95	Total phosphorus (P)	0.64 - 0.69%
Salt	0.32	Available phosphorus (AP)	0.36 - 0.41%
Fish meal	2.9	Lysine (Lys)	1.02 - 1.08%
Core material	1.05	Methionine (Met)	0.41 - 0.46%
Choline chloride	0.06	Threonine (Thr)	0.69 - 0.74%
Threonine	0.28	Sodium (Na)	0.16%
Methionine	0.32	Vitamin A	11800 - 12200 IU/kg
Lysine	0.18	Vitamin D3	2900 - 3100 IU/kg
98% lysine	0.29	Vitamin E	25 - 50 mg/kg
Vitamin premix	0.05		
